# Single arm prospective multicenter case series on the use of burst stimulation to improve pain and motor symptoms in Parkinson’s disease

**DOI:** 10.1186/s42234-020-00055-3

**Published:** 2020-09-28

**Authors:** Krishnan V. Chakravarthy, Rahul Chaturvedi, Takashi Agari, Hirokazu Iwamuro, Rajiv Reddy, Ayano Matsui

**Affiliations:** 1 Division of Pain Medicine, Department of Anesthesiology, University of California San Diego Health Center, 9400 Campus Point Dr, La Jolla, San Diego, CA USA; 2 VA San Diego Health Care, 3350 La Jolla Village Dr, San Diego, CA USA; 3 Department of Neurosurgery, Tokyo Metropolitan Neurological Hospital, Tokyo, Japan; 4 Department of Research and Therapeutics for Movement Disorders, Juntendo University Graduate School of Medicine, Tokyo, Japan; 5 Department of Orthopedics, National Center Hospital of Neurology and Psychiatry, Tokyo, Japan

**Keywords:** Burst simulation, Parkinson disease, Spinal cord stimulation, Dorsal column stimulation, Deep brain stimulation

## Abstract

**Background:**

In this study we analyze new clinical data in the use of spinal cord stimulation (SCS) for the treatment of pain and motor symptoms in patients with Parkinson’s Disease (PD), as both a singular bioelectric therapy and as a salvage therapy after deep brain stimulation (DBS).

**Methods:**

Fifteen patients were recruited and had percutaneous electrodes implanted at the level of the thoracic or cervical spine. Participants were set to one of three stimulation modes: continuous tonic stimulation, continuous Burst stimulation (40 Hz, 500 Hz, 1000 μs), or cycle mode (on time of 10–15 s, off time of 15–30 s) with Burst (40 Hz, 500 Hz, 1000 μs). Patients completed the Visual Analogue Scale (VAS), Unified Parkinson’s Disease Rating Scale, Self-Rating Depression Scale, Hamilton Depression Rating Scale, Profile of Mood State, 10-meter walking test, and the Timed Up and Go (TUG).

**Results:**

All patients experienced significant improvement in VAS scores with a mean reduction of 59% across all patients. Patients who chose the cycling burst stimulation parameter had an average 67% reduction in VAS scores, as compared to the continuous burst parameter group, which had an average 48% reduction in VAS scores. Seventy-three percent of patients experienced improvement in the 10-meter walk, with an average improvement of 12%. Sixty-four percent of patients experienced clinically relevant improvements in the TUG, with an average improvement of 21%.

**Conclusions:**

This study points to the potential utility of SCS to address both pain and certain aspects of motor symptoms in PD patients who have and have not received DBS therapy.

## Background

Parkinson’s disease (PD) is a progressive multi-system, neurodegenerative disease that leads to both motor and non-motor symptoms (Sveinbjornsdottir, 2016; Kalia & Lang, 2015). The most common motor symptoms include tremor, bradykinesia, rigidity, and postural instability. Non-motor symptoms include pain, orthostatic hypotension, urinary disturbances, sleep disorders, and various neuropsychiatric symptoms. Both sets of symptoms have significant impact on PD patients’ quality of life and mortality (Sveinbjornsdottir, 2016; Sauerbier et al., 2016; Martinez-Martin, 2011). The wide array of PD symptoms has been shown to alter family relationships, lead to a loss of self-identity, and contribute toward a sense of being deprived of one’s self-worth (Sjödahl Hammarlund et al., 2018). Therapeutic options aimed at alleviating PD symptoms are thus vital for disease management.

Though the exact cause of PD continues to be studied today, there exist two governing markers underlying the pathophysiology of the disease process. These include the degeneration of dopaminergic neurons in the nigrostriatal pathway, and the presence of intracytoplasmic proteinaceous inclusion bodies in surviving neurons, referred to as Lewy bodies. The degeneration of dopaminergic neurons in the nigrostriatal pathway is thought to reduce inhibition of the thalamus and decrease excitatory input to the motor cortex, ultimately leading to bradykinesia and other PD symptoms (Blandini, 2013; Dauer & Przedborski, 2003). The decrease in dopaminergic neural firing in the nigrostriatal pathway may disrupt the neural oscillations in the basal ganglia. Specifically, it may lead to increased firing of striatal neurons in the indirect pathway, with the recruited neurons firing with an excess of beta (13–30 Hz) oscillations in the motor system (Beudel et al., 2019). The two mainstay therapies of PD, dopamine and deep brain stimulation (DBS), have both been shown to alter the pathological changes in electrical oscillations (Kühn et al., 2006; Eusebio et al., 2011).

Though dopamine and DBS are the gold standard treatment for PD, they both continue to have severe limitations and side effects to consider. For dopamine treatment, side effects include dyskinesias, GI disturbances, orthostatic hypotension, and neuropsychiatric features including anxiety and hallucinations (Connolly & Lang, 2014). While it is possible to adjust dosing parameters to ameliorate some of the side effects above, one of the more prominent issues is that dopamine agonists are associated with loss of efficacy over time (LeWitt et al., 2019). DBS has been shown to have excellent outcomes in alleviating some of the motor and non-motor symptoms of PD (Okun, 2012; De Hemptinne et al., 2015; Fasano et al., 2012). However, there are several associated risks with DBS, including intracranial bleeding (up to 5.0%), hardware issues, infection, incorrect placement, mis-positioning, and seizures (up to 2.4%) (Okun, 2012; Larson, 2014). The risk of infection has been reported to range from 1.2 to 15.2% (Okun, 2012). Similar to dopamine treatment, the use of DBS may have decreased efficacy over time as well (Paschen et al., 2019).

An alternate therapy that can be used to alleviate both motor and non-motor symptoms of PD is spinal cord stimulation (SCS) technology. Spinal cord stimulation of the dorsal columns within the epidural space has already been shown to be beneficial in a multitude of pain conditions (Jeon, 2012; Verrills et al., 2016). SCS has been shown to stimulate large non-nociceptive myelinated fibers of the peripheral nerves (A-β fibers), leads to inhibition of the small nociceptive projections (A-δ and C) in the dorsal horn. Additionally, SCS may lead to the release of GABA, substance-P and serotonin, neurotransmitters involved in pain modulation (Jeon, 2012). The new therapy has also been shown to improve the motor symptoms of PD (de Andrade et al., 2016; Samotus et al., 2018). Thus, SCS may be an excellent therapeutic option to alleviate both motor symptoms and non-motor symptoms such as pain in PD patients. Whether to use SCS as a singular bioelectric therapy option or as a salvage therapy after dopamine and DBS treatments have begun to lose efficacy, continues to be a question of interest. There have been studies that have shown SCS to be a reasonable salvage option after dopamine and DBS (Pinto de Souza et al., 2017). However, with any therapy the risks must be considered. Risks for SCS include undesirable feelings such as a jolt or shock, hematoma, infection, seroma, and in extreme cases, epidural hemorrhage (Medtronic, 2020). The data thus far point to SCS as being a viable alternative, conjunctive and or potential salvage therapy for those with PD. This paper aims to analyze clinical translational data for SCS in PD as both singular bioelectric therapy and salvage therapy after loss of efficacy of DBS for both motor and non-motor symptoms such as pain.

## Methods

### Subjects

In this non-randomized study, a total of 15 PD patients were recruited through a convenience sampling method, whose pain was refractory to medical therapy and other conservative treatments. The etiology of pain was determined by history and neurological physical findings, imaging diagnosis using MRI and CT, and responsiveness to nerve block and medications. The study was approved by the Institutional Review Boards at each respective center. The PI and sub-investigators referred subjects to the study from their own practices, if the patients were eligible to be implanted with the SCS system and were interested in participation. Patients were included in the study if they had chronic pain of predominantly neuropathic origin that was refractory to conventional treatments such as analgesic drugs and nerve blocks. In addition, patients were eligible if their pain had not improved even with adequate dopaminergic drug administration and adjustment of DBS parameters (if the patients had previously received DBS). Most patients had fluctuating pain in conjunction with motor fluctuations. For example, the patients’ pain increased when they were off dopaminergic drugs and/or when DBS was turned off. Overall, the causes of pain in the patients enrolled in this study were as follows: 5 cases after lumbar spine surgery, 4 cases of radicular pain due to compression fractures (spinal surgery was not indicated because of severe osteoporosis or spinal scoliosis), 1 case of radicular pain due to severe spinal scoliosis, 3 cases of postural abnormalities (in this group pain occurred by standing for a long time with scoliosis and bent posture), and 2 cases with unknown etiology (Table 1). Exclusion criteria included: 1) active suicidal ideation, 2) substance abuse/addiction history, 3) chronic illness / cancer diagnosis, 4) life-threatening illness, 5) implanted devices such as a cardiac pacemaker, and 6) participating in another clinical study. DBS was not included in the exclusion criteria.

**Table 1:** Demographic Information

N	Average age	Average PD duration (Y)	Type of PD initially	Indication for SCS	DBS prior	Leads and location	Follow up period (months)
15	74	17	Tremor Dominant: 2	Pain multiple sites: 7	No: 7	Thoracic: 14	Range: 4–33
			Akinetic Rigid: 13	Low back pain only: 6	Yes: 8	Cervical: 1	Average: 22
				Leg Pain: 2			

### SCS intervention

A total of 15 patients were included in the study with a mean age of 74 (SD 5.2) and an average PD duration of 17 years (SD 8.7) (Table 1). All participants had a history of concurrent pain conditions. Seven patients had no DBS therapy prior to the study, while 8 had undergone DBS prior to initiation. One or two percutaneous electrodes were implanted in the epidural space on the dorsal midline at the level of the thoracic or cervical spine and connected to an implantable pulse generator (Abbott Proclaim Elite5 or Abbott Prodigy); 1 Abbott Lamitrode lead in 5 patients, 1 Abbott Octrode lead in 1 patient, 2 Abbott Octrode leads in 3 patients, 1 Abbott Octrode lead with 1 Abbott Lamitrode lead in 4 patients, and 2 Medtronic Octad leads in 2 patients, in which a Medtronic IPG was previously used and changed to an Abbott IPG with a conversion connector. Lead choice (e.g, company and type of lead used) was determined based on differing protocols of the Japanese hospital systems. Participants were set to one of three stimulation modes according to their preference: the continuous tonic stimulation, the continuous Burst stimulation (40 Hz, 500 Hz, 1000 μs), or the cycle mode (on time of 10–15 s, off time of 15–30 s) with Burst (40 Hz, 500 Hz, 1000 μs). While traditional spinal cord stimulation has been delivered in a tonic fashion, the majority of patients in this study opted for the relatively newer, “burst SCS” in either a continuous or cycle mode timeframe. The burst SCS stimulation, demonstrated by De Ridder, is characterized by a five-pulse train with an intraburst frequency of 500 Hz, with a 40 Hz passive recharging model. In order to ensure proper electrode placement, a low-frequency tonic stimulation was first applied to induce a paresthesia or alternate sensory experience such as tingling in the primary area of the patient’s pain (e.g, lower back). Next, the stimulation waveform was changed to the stimulation parameter that the patient had chosen (e.g, burst stimulation, burst stimulation with cycle mode, tonic stimulation). The intensity of the stimulation was set to 60–70% of the threshold for paresthesias evoked by burst during intraoperative testing. All patients did not feel stimulation-induced paresthesias after the intensity was reduced to 60–70% of the threshold for paresthesias, except for one patient who chose either the continuous tonic (2.6 mA, 10 Hz, 350 μs) or the continuous Burst (0.15 mA) stimulation based on his severity of pain. The patients had different levels of pain that were altered by the action of both dopaminergic drugs and DBS. Therefore, the effects of SCS were evaluated “on-medication” and “on-DBS” under the condition that the dopaminergic drugs and DBS were sufficiently controlled so that there were no off-period painful sensations. The careful and adequate administration of dopaminergic drugs (or DBS programming) prevented the appearance of off-period painful sensations and controlled other motor and non-motor symptoms in Parkinson’s disease. In total, there were 8 patients who had previously received DBS and 7 patients who received only drug treatment (anti-Parkinsonian drugs and other agents for neuropathic pain). All patients underwent bilateral subthalamic deep brain stimulation (STN-DBS). Patients who received DBS were combined with anti-Parkinson drugs medication. In patients who received DBS, the therapeutic effect of SCS was evaluated at “on-medication” and “on-DBS”. In patients who received only drug therapy, the therapeutic effect of SCS was evaluated in the “on-medication” state. The exact configuration for each patient and stimulation parameters can be found in Table 2.

**Table 2:** Lead locations and stimulation parameters

Patient	DBS prior	Lead locations	Stimulation parameter
1	No	T7-T9 T10-T12	Proclaim Elite5 (Abbott), 5–6-(at T8 level) 9-(at T10 level) 15 + 16+ (at T12 level), 0.5 mA Burst (40 Hz, 500 Hz, 1000us), Cycle mode (on time 15 s, off time 15 s)
2	No	T8–9 T10–11	Prodigy (Abbott), 3–8+, 0.5 mA, Burst (40 Hz, 500 Hz, 1000us)
3	No	C2-C5	Proclaim Elite5 (Abbott), 2–4+, 0.4 mA, Burst (40 Hz, 500 Hz, 1000us), Cycle mode (on time 30s, off time 90 s)
4	No	T6-T8	Proclaim Elite5 (Abbott), 3–4-12 + 13+, 0.8 mA, Burst (40 Hz, 500 Hz, 1000us)
5	No	T10-T11 T11-T12	Proclaim Elite5 (Abbott), 15–16-(at T12 level) 11 + 12+ (at T10 level) 0.15 mA, Burst (40 Hz, 500 Hz, 1000us)
6	No	T8-T10	Proclaim Elite5 (Abbott), 2–10-(at T8/9 level) 3 + 11+ (at T9 level) 0.15 mA, Burst (40 Hz, 500 Hz, 1000us)
7	No	T9-T11	Proclaim Elite5 (Abbott), 2–4 + (at T9/10 level) 2.6 mA, tonic 2.6 mA 10 Hz 350us or 0.15 mA, Burst (40 Hz, 500 Hz, 1000us), (Switching from Burst mode to tonic SCS mode when severe pain is occurred)
8	Yes	T8-T9 T10-T11	Proclaim Elite5 (Abbott), 1–2-7 + 8+, 1.45 mA, Burst (40 Hz, 500 Hz, 1000us), Cycle mode (on time 10s, off time 30 s)
9	Yes	T7-T9	Proclaim Elite5 (Abbott), 6–7 + 13–14-15 + 16+, 0.6 mA, Burst (40 Hz, 500 Hz, 1000us), Cycle mode (on time 10s, off time 30 s)
10	Yes	T9-T10	Proclaim Elite5 (Abbott), 3–2 + 4+ (at T9/10 level), 0.40 mA, Burst (40 Hz, 500 Hz, 1000us), Cycle mode (on time 30s, off time 90 s)
11	Yes	T9-T10	Proclaim Elite5 (Abbott), 2–1 + 3+ (at T9 level), 0.20 mA, Burst (40 Hz, 500 Hz, 1000us)
12	Yes	T6-T9	Proclaim Elite5 (Abbott),10–12+ (at T7 level), 0.80 mA, Burst (40 Hz, 500 Hz, 1000us), Cycle mode (on time 30s, off time 90 s)
13	Yes	T5-T7 T9-T11	Proclaim Elite5 (Abbott),11–10 + 12+ (at T9/10 level), 3.0–4.5 mA, tonic 40 Hz 350us
14	Yes	T2-T4	Proclaim Elite5 (Abbott),4–5-6 + 7+ (at T3 level), 0.75 mA, Burst (40 Hz, 500 Hz, 1000us)
15	Yes	T9-T10 T10-T11	Proclaim Elite5 (Abbott),11–13+ (at T9/10 level), 0.35 mA, Burst (40 Hz, 500 Hz, 1000us), Cycle mode (on time 15 s, off time 45 s)

### Clinical outcomes

Patients were asked to complete the Visual Analogue Scale for pain (VAS), the revised Unified Parkinson’s Disease Rating Scale (MDS-UPRS), Self-Rating Depression Scale (SDS), Hamilton Depression Rating Scale (HAMD), Profile of Mood State (POMS-II), 10 M (10-meter) walking test, and a Timed Up and Go (TUG) at each clinic visit. Each participant had a family member or close friend present during clinic visits to help answer questionnaires.

### Statistical analysis

Mean and standard deviations were calculated for two separate groups, those who had DBS prior to entering the study, and those that did not. Paired two-tailed t-tests were calculated comparing pre-intervention means and post-intervention means within each group (Table 3). As a sensitivity analysis, we also performed a Wilcoxon sign rank test for certain parameters.

**Table 3:** Outcome measures pre and post stimulation therapy

Outcome	DBS prior / stimulation parameter	Pre stimulation (SD)	Post stimulation (SD)	*P*-value^1^	Sample size (n)
VAS scores	No	8.9 (1.4)	3.8 (2.6)	0.0007^2^	7
	Yes	8.5 (1.4)	3.3 (2.5)	.0001^2^	8
VAS scores	Continuous burst	9.4 (0.8)	4.9 (2.5)	0.002	6
	Cycle mode burst	7.8 (1.4)	2.6 (2.0)	.0001	7

^1^**(**t-test, two-tailed**)**

^2^Significant value defined as *p*-value < 0.05

## Results

**Fig. 1:**
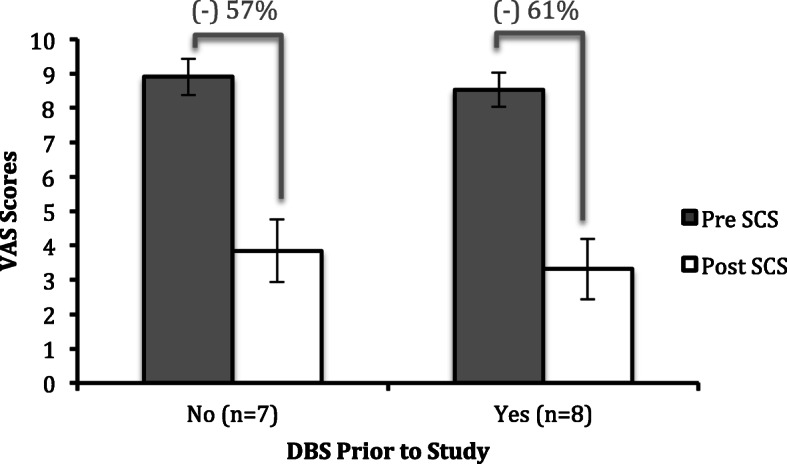
Pre and Post VAS Scores Stratified by DBS Prior to Study

**Fig. 2:**
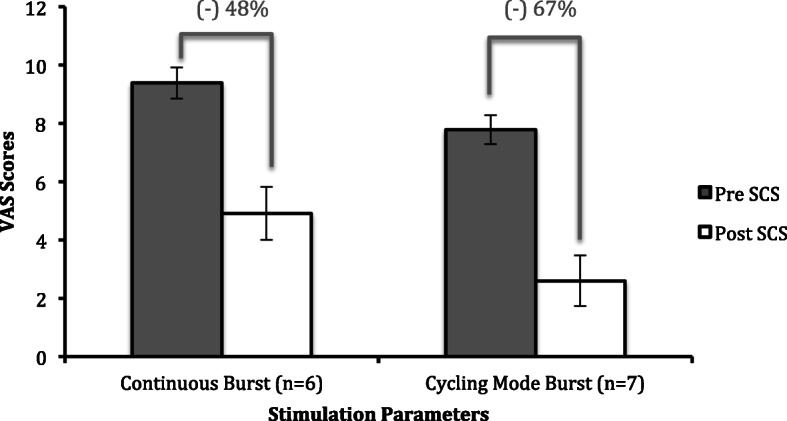
Pre and Post VAS Scores Stratified by Stimulation Parameters

All patients in the study experienced significant improvement in VAS pain scores after implantation of SCS (two-tailed t-test, *p* < 0.005) (Table 3, Fig. 1). Specifically, for patients who did not receive DBS prior to SCS, the average percent reduction was 57% (two-tailed t-test, *p* < 0.0007), and for those who did receive DBS, the average percent reduction was 61% (two-tailed t-test, *p* < 0.0001). As a sensitivity analysis, we also performed a Wilcoxon sign ranked test, which also found a *p*-value of < 0.05. Additionally, we found that those patients who chose the cycling burst stimulation parameter had an average 67% reduction in VAS scores, as compared to the continuous burst parameter group, which had an average 48% reduction in VAS scores (Fig. 2). The effects of SCS for neuropathic pain did not differ between the “Meds+SCS” group and the “Meds+DBS + SCS” group. When comparing the mean differences for the 10-meter walk and the TUG before and after SCS therapy, there was no statistically significant difference between groups (Table 3). However, out of the 11 total patients who were able to complete the 10-meter walk before and after therapy, 8 of them (73%) showed improvement in their completion time, with an average improvement of 12% (two-tailed paired t-test, *p* = 0.003) (Supplementary Figure 1). Out of the 11 patients who completed the TUG, 7 of them (64%) showed clinically significant improvement in their completion time with an average improvement of 21% (two-tailed paired t-test, *p* = 0.03) (Supplementary Figure 2). When stratifying by stimulation parameters, those who chose a continuous burst pattern had an 18% improvement in TUG scores, while those who chose cycling mode burst stimulation had a 7% worsening in scores (Supplementary Figure 3). MDS-UPRS, SDS, and Hoehn and Yahr scores did not differ between pre and post SCS stages. For those that had not receive DBS prior to the study, HAMD and POMS-II scores increased, while for those who had received DBS prior, both HAMD and POMS-II scores decreased (Supplementary Table 1). However, given the low number of patients who fully completed these assessments, the standard deviation for each of these parameters was relatively high.

## Discussion

Motor symptoms such as tremor, bradykinesia, rigidity, and postural instability, as well as concurrent pain symptoms significantly impact PD patients’ quality of life. SCS is an emerging technology that can potentially be utilized to treat both the motor and non-motor symptoms such as pain that patients with PD deal with on a daily basis. There are many theories as to how burst SCS leads to an improvement in motor function in patients with PD, and there continues to be ongoing research in the area. While some neurons within the dorsal column generate single action potentials, other neurons in the region fire in a burst of action potentials. These bursts are thought to lead to variations in downstream modulation of the lateral and medial spinothalamic tracts (Chakravarthy et al., 2019). In a preclinical study involving rat models, Remy and Spruston delivered a single burst stimulation to the Schaffer collateral pathway in the hippocampus, which produced long term potentiation (LTP) at the neural synapse between the collaterals and postsynaptic CA1 neurons (Remy & Spruston, 2007). This study showed the potential for burst SCS to produce long lasting changes to neural networks involved in the pain networks of the central nervous system. It has also been shown through animal models that burst SCS, unlike tonic SCS, does not evoke alteration in the dorsal column nuclei, such as the gracile nucleus, a possible mechanism for reduced paresthesias seen with burst SCS. Preclinical studies have also shown burst SCS to depress wide dynamic range (WDR) neurons within the dorsal horns, which may be a key component in SCS’s mechanism for pain reduction (Crosby et al., 2015). Clinically, SCS has been thought to stimulate the superficial fibers of the dorsal column, which in turn can lead to the increased release of dopamine (Fuentes et al., 2010; Shon et al., 2010). Other theories suggest that SCS can lead to a more neuroprotective role, decreasing the rate of dopaminergic degeneration (Fuentes et al., 2010; Gubellini et al., 2009). Falowski et al. measured somatosensory evoked potentials (SSEPs) from different types of SCS waveforms (e.g. tonic, burst etc) and found that burst stimulation inhibited somatic sensory transduction, and tended to activate distal muscles from the site of stimulation at lower amplitudes and proximal muscles with higher amplitudes (Falowski, 2019). The group found the opposite finding for lower frequency tonic stimulation, indicating that burst SCS and tonic SCS differ in modulation of fibers within the dorsal column.

There have been very few studies that have tested whether SCS can lead to improvement in motor function and concurrent pain in patients with PD. A previous case report showed that SCS placed in the thoracic epidural space (T9–10) level, led to improvement in motor function in PD patients. In the current study, 14/15 subjects had SCS placed in the thoracic epidural space. While the mean differences between pre SCS implantation and post SCS implantation groups for motor function (10 M walk, TUG) were not statistically different, most patients that were able to complete the tests showed improvement in their completion times for both tasks. Additionally, after removal of outliers, there was a statistically significant improvement in motor function for both groups (Table 3). However, given the study design, it is not possible to differentiate whether these motor improvements stemmed from the effects of SCS, or if the decrease in pain allowed patient to improve their motor testing results. Thus, additional studies need to be conducted to explore the efficacy of SCS placed in the thoracic epidural space to help alleviate PD motor symptoms, and to explore the causal relationships between the SCS, pain and motor improvement.

SCS could be utilized even after DBS treatment in our study and led to significant pain relief in PD patients. SCS after failed DBS therapy was also able to help a subset of patients in our cohort with motor symptoms, though the difference between the groups as a whole did not show statistical significance. Future studies should be conducted that analyze the use of SCS as a salvage therapy for Parkinson’s disease symptoms after failed DBS therapy.

There are several limitations to this study. The patients did not receive the spinal cord stimulator in the exact same spinal location due to differences in presenting pain symptoms. Additionally, not every patient was able to return and complete the TUG and 10 M walk test, which decreased the sample size. However, the majority of patients still had leads placed in the thoracic spine, and the majority of patients were able to complete the questionnaires and tests given in the study. We believe that the data in this study can be helpful to guide future studies that utilize SCS as salvage therapy for Parkinson’s disease to improve the body of literature on the use of SCS in PD patients.

## Conclusions

In this study, all patients showed a statistically significant improvement in VAS pain scores after receiving SCS. The majority of patients also showed improvements in motor functioning after SCS even after failed DBS therapy. This study thus points to the potential utility of SCS as an option to address both pain and motor symptoms in PD patients who have and have not received DBS therapy.

## Supplementary information

**Additional file 1:** **Supplementary Figure 1.** Pre and Post SCS Result for 10 Meter Walk in Patients who Responded

**Additional file 2:** **Supplementary Figure 2.** Pre and Post Spinal Cord Stimulation TUG Results

**Additional file 3:** **Supplementary Figure 3.** Pre and Post Stimulation TUG Scores Stratified by Stimulation Parameters

**Additional file 4:** **Supplementary Table 1.** Other Clinical Characteristics Pre and Post Stimulation

## Data Availability

The datasets used and/or analysed during the current study are available from the corresponding author on reasonable request.

## Abbreviations

PD: Parkinson’s Disease
DBS: Deep brain stimulation
VAS: Visual Analogue Scale
TUG: Timed Up and Go
SCS: Spinal cord stimulation
MDS-UPRS: Unified Parkinson’s Disease Rating Scale
SDS: Self-Rating Depression Scale
HAMD: Hamilton Depression Rating Scale
POMS-II: Profile of Mood State
10 M: 10 M (10-meter) walking test
WDR: Wide dynamic range
SSEPs: Somatosensory evoked potentials
