# Supplementary figures and images for: Single arm prospective multicenter case series on the use of burst stimulation to improve pain and motor symptoms in Parkinson’s disease
Source: Bioelectron Med. 2020 Sep 28;6:18. doi: 10.1186/s42234-020-00055-3 (PMC7520952; doi:10.1186/s42234-020-00055-3)

Supplementary Figure 1. Pre and Post SCS Result for 10 Meter Walk in Patients who Responded

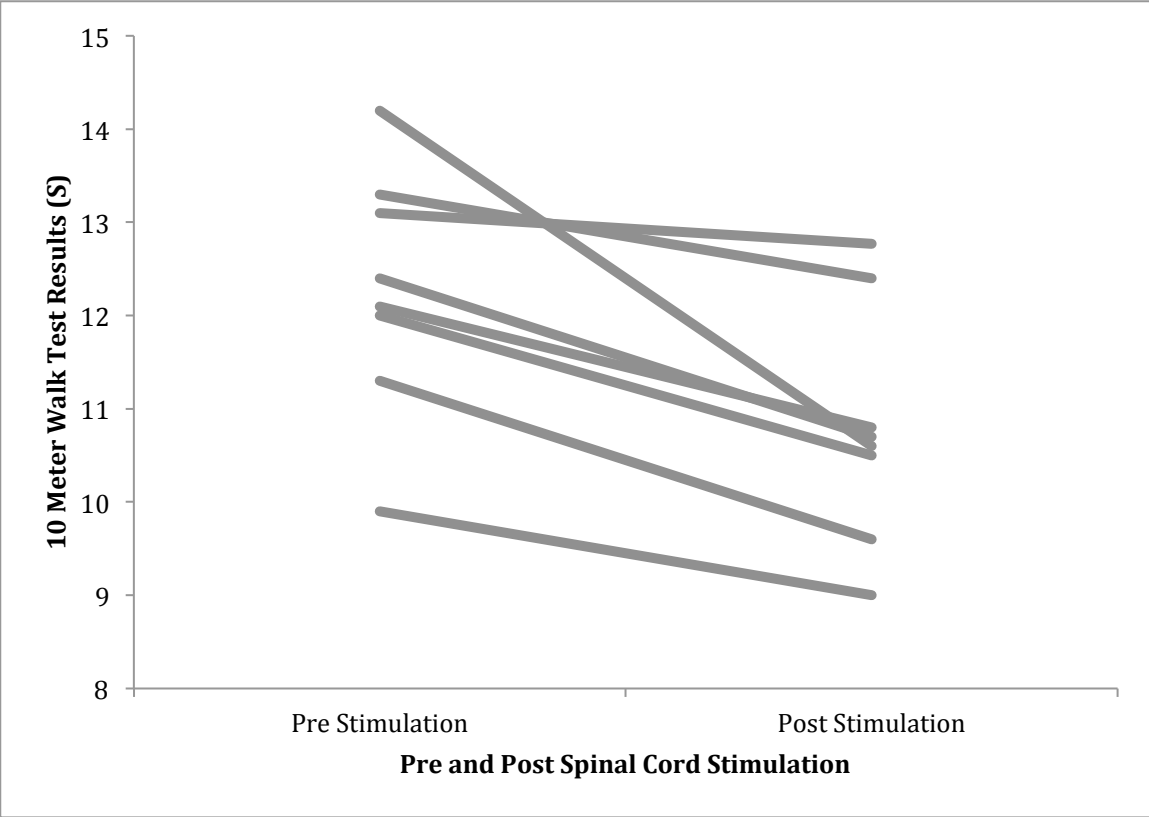

Supplement: Supplementary file 1 — Additional file 1: Supplementary Figure 1. Pre and Post SCS Result for 10 Meter Walk in Patients who Responded [file 42234_2020_55_MOESM1_ESM.pdf]

Supplementary Figure 2. Pre and Post Spinal Cord Stimulation TUG Results

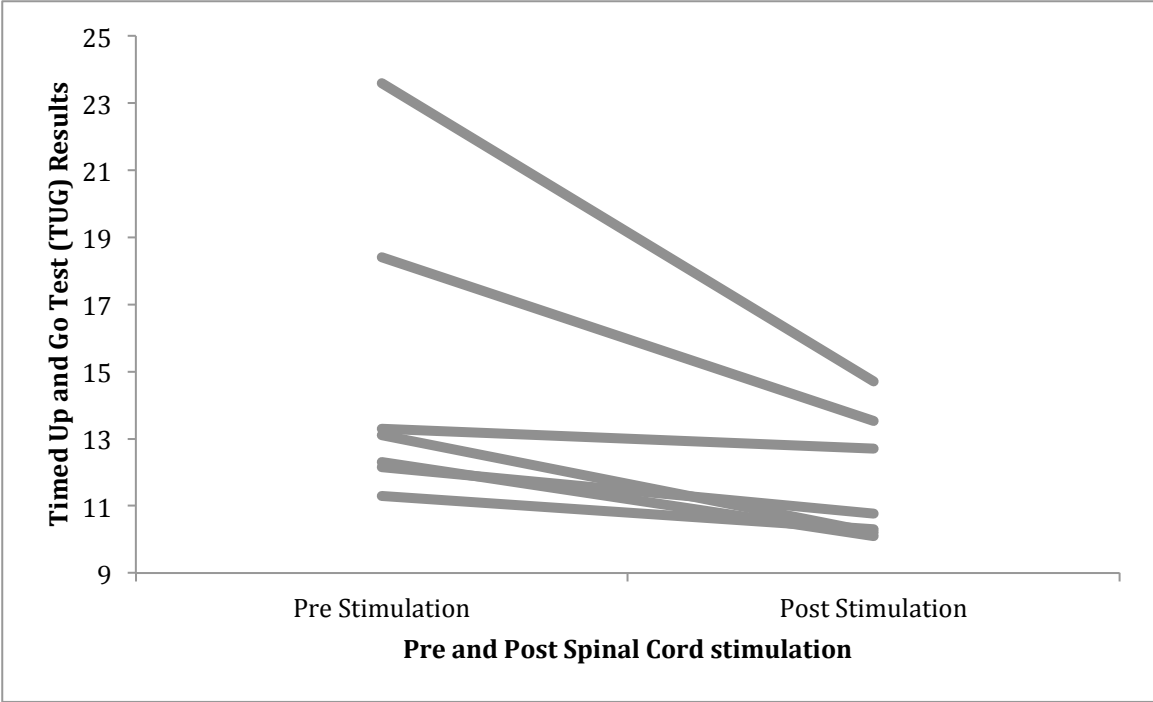

Supplement: Supplementary file 2 — Additional file 2: Supplementary Figure 2. Pre and Post Spinal Cord Stimulation TUG Results [file 42234_2020_55_MOESM2_ESM.pdf]
