# Supplementary material for: Single arm prospective multicenter case series on the use of burst stimulation to improve pain and motor symptoms in Parkinson’s disease
Source: Bioelectron Med. 2020 Sep 28;6:18. doi: 10.1186/s42234-020-00055-3 (PMC7520952; doi:10.1186/s42234-020-00055-3)

Supplementary Figure 3. Pre and Post Stimulation TUG Scores Stratified by Stimulation Parameters

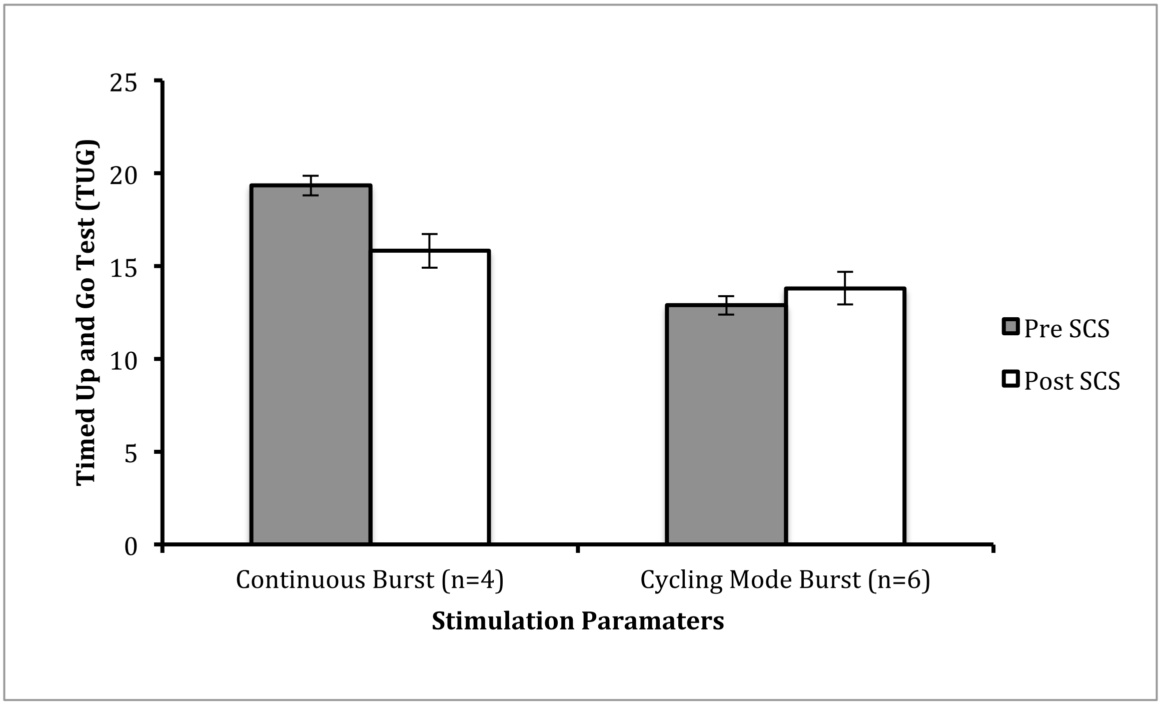

Supplement: Supplementary file 3 — Additional file 3: Supplementary Figure 3. Pre and Post Stimulation TUG Scores Stratified by Stimulation Parameters [file 42234_2020_55_MOESM3_ESM.pdf]
