# Supplementary material for: Single arm prospective multicenter case series on the use of burst stimulation to improve pain and motor symptoms in Parkinson’s disease
Source: Bioelectron Med. 2020 Sep 28;6:18. doi: 10.1186/s42234-020-00055-3 (PMC7520952; doi:10.1186/s42234-020-00055-3)
Supplement: Supplementary file 4 — Additional file 4: Supplementary Table 1. Other Clinical Characteristics Pre and Post Stimulation [file 42234_2020_55_MOESM4_ESM.docx]

Supplementary Table 1. Other Clinical Characteristics Pre and Post Stimulation

| **Outcome** | **DBS Prior** | **Pre Stimulation (SD)** | **Post Stimulation (SD)** | **Sample Size (n)** |
| --- | --- | --- | --- | --- |
| Hoehn and Yahr | No | 4.3 (0.5)  3.8 (0.5) | 4.4 (0.5) | 7 |
|  | Yes |  | 3.9 (0.7) | 8 |
| UPDRS | No | 35.8 (12.6) | 35.3 (12.6) | 4 |
|  | Yes | 23.9 (7.3) | 23.5 (7.1) | 8 |
| SDS | No | 49.0 (7.8) | 49.0 (9.5) | 6 |
|  | Yes | 54.5 (4.7) | 53.6 (7.5) | 5 |
| HAMD | No | 5.0 (1.0) | 8.7 (3.5) | 3 |
|  | Yes | 9.5 (9.5) | 4.8 (3.6) | 4 |
| POMS-2 | No | 48.0 (5.7) | 53.0 (2.8) | 2 |
|  | Yes | 50.3 (9.3) | 47.8 (9.9) | 4 |
| *Note, All variables were recorded while patients were on medications and on DBS | | | | |
